# Advantages of Chinese herbal medicine in treating rheumatoid arthritis: a focus on its anti-inflammatory and anti-oxidative effects

**DOI:** 10.3389/fmed.2024.1371461

**Published:** 2024-03-07

**Authors:** Xiaoyu Wang, Youqian Kong, Zeguang Li

**Affiliations:** ^1^Graduate School, Heilongjiang University of Chinese Medicine, Harbin, China; ^2^First Affiliated Hospital, Heilongjiang University of Chinese Medicine, Harbin, China

**Keywords:** traditional Chinese medicine, Chinese herbal medicine, rheumatoid arthritis, oxidative stress, inflammation

## Abstract

Oxidative stress is a condition characterized by an imbalance between the oxidative and antioxidant processes within the human body. Rheumatoid arthritis (RA) is significantly influenced by the presence of oxidative stress, which acts as a pivotal factor in its pathogenesis. Elevated levels of mitochondrial reactive oxygen species (ROS) and inflammation have been found to be closely associated in the plasma of patients with RA. The clinical treatment strategies for this disease are mainly chemical drugs, such as nonsteroidal anti-inflammatory drugs (NSAIDs), disease-modifying anti-rheumatic drugs (DMARDs), glucocorticoids (GCs) and biological agents, but it is difficult for patients to accept long-term drug treatment and its side effects. In the theory of traditional Chinese medicine (TCM), RA is thought to be caused by the attack of “wind, cold, damp humor,” and herbs with the effect of removing wind and dampness are used to relieve pain. Chinese herbal medicine boasts a rich heritage in effectively attenuating the symptoms of RA, and its global recognition continues to ascend. In particular, RA-relevant anti-inflammatory/anti-oxidative effects of TCM herbs/herbal compounds. The main aim of this review is to make a valuable contribution to the expanding pool of evidence that advocates for the incorporation of Chinese herbal medicine in conventional treatment plans for RA.

## 1 Introduction

RA is a persistent autoimmune condition that is characterized by widespread inflammation throughout the body. It primarily affects the joints, causing pain, stiffness, and swelling. The prevalence of RA varies globally, with a higher incidence rate in women compared to men. The worldwide prevalence ranges from 0.5 to 1.0%. RA is characterized by the presence of neovascularization and abnormal synovial hyperplasia, which are pathological manifestations directly influenced by the infiltration of diverse immune and inflammatory cells within the affected area (1, 2). The infiltration of the affected area at a local level ultimately results in the gradual deterioration of both cartilage and bone, thus causing dysfunction of the joints and posing a higher risk of disability and even mortality. Oxidative stress is a condition characterized by an imbalance between the oxidative and antioxidant processes within the human body. The disruption in redox balance gives rise to an excess of free radicals, which surpasses the body’s ability to eliminate them through antioxidants. As a result, an excess of ROS, reactive nitrogen species (RON), and various other compounds build up, leading to oxidative damage. RA is significantly influenced by the presence of oxidative stress, which acts as a pivotal factor in its pathogenesis (3, 4). The research has uncovered a noteworthy and affirmative association between clinical indicators and oxidative stress indicators within the bloodstream of individuals suffering from RA. The measurement of serum oxidative stress markers has proven to be a reliable biomarker for effectively monitoring the progression of RA disease (4, 5). Elevated levels of mitochondrial ROS and inflammation have been found to be closely associated in the plasma of patients with RA. The treatment involving tumor necrosis factor (TNF) blockade has the capacity to inhibit oxidative stress and the occurrence of mitochondrial mutations induced by hypoxia, which in turn facilitates the process of disease rehabilitation (6).

At present, the clinical treatment strategies for this disease are mainly chemical drugs, such as NSAIDs, DMARDs, GCs and biological agents, but it is difficult for patients to accept long-term drug treatment and its side effects. Nowadays, the clinical acceptance of TCM has been increasing all over the world. In the theory of TCM, RA is thought to be caused by the attack of “wind, cold, damp humor,” and herbs with the effect of removing wind and dampness are used to relieve pain (7, 8). Wind in TCM is characterized by the sudden onset of illness, mobility of the affected area, variability in symptom presentation, and sensitivity to environmental changes. Wind-dominant arthralgia is commonly seen in the initial phase of RA and primarily affects the upper body. Damp is primarily associated with the weather. Being in water or resting on damp ground can trigger and exacerbate symptoms due to the moist surroundings. Cold-induced symptoms refer to the exacerbation of symptoms when exposed to cold temperatures, which can be alleviated by the application of heat. Cold-related arthritis primarily affects the hands and feet. Chinese herbal medicine can alleviate RA through multi-target, multi-link and multi-way, and concentrate on the regulation of oxidative stress and inflammation, both of which play pivotal roles in the progression of RA (8, 9). The objective of this research is to explore the RA-relevant anti-inflammatory/anti-oxidative effects of TCM herbs/herbal compounds. Through emphasizing the distinctive advantages of Chinese herbal medicine in dealing with these fundamental pathological processes, our objective is to shed light on potential innovative approaches to improve RA patient outcomes. Ultimately, the primary goal of this paper is to make a valuable contribution to the expanding pool of evidence that advocates for the incorporation of Chinese herbal medicine in conventional treatment plans for RA.

## 2 The pathogenesis of rheumatoid arthritis

RA is initially characterized by persistent activation of cells, leading to autoimmunity in the joints or other affected organs (10). The primary presentation of the illness primarily arises as a result of inflammation in the synovial tissue and damage to the joints. Despite the absence of a cure for RA, early intervention with medication can effectively decrease the likelihood and severity of joint damage, while also slowing the progression of this debilitating disease. In the field of clinical treatment for RA, commonly used medications include NSAIDs, GCs, and DMARDs.

RA is characterized by a sophisticated interplay between genetic factors and environmental triggers (11). Over the course of recent decades, a substantial body of evidence has unequivocally demonstrated the pivotal role of genetic factors in triggering RA. At present, the genes HLA-DRB1, TNFRSF14, and PTPN22 have been identified as genetic factors that contribute to the development of RA, indicating a strong association between these genes and the onset of RA. Antigen-presenting cells have the capability to mistakenly present their antigens to T cells, a process that triggers T cell-mediated autoimmune reactions and directly contributes to the development of RA (12, 13). Moreover, RA is significantly influenced by various environmental factors (14). Smoking, individual dietary choices, and personal hygiene practices are among the major contributors (14, 15). These factors have a direct impact on the post-transcriptional modifications of specific genes and can also indirectly affect susceptible genes through epigenetic mechanisms.

Rheumatoid factor (RF) isotypes in combination with anti-cyclic citrullinated peptide 2 (anti-CCP2) antibodies yielded higher risk ratios for disease development than each factor separately, suggestive of an interaction between RFs and anti-cyclic citrullinated peptide antibody (ACPA) (16). IgM-RF enhanced the capacity of ACPA immune complexes to further stimulate cytokine production by macrophages, and consequently that RF would affect the immune process and/or the pathogenicity of ACPA immune complexes in RA (17, 18). Researches indicate that there exist two distinct genetic types of RA, known as ACPA positive and ACPA negative, which exhibit varying degrees of association and shared epitopes among patients (11, 19). When specific alterations occur within the surrounding conditions, the arginine undergoes a conversion into citrulline, initiated by the enzymes known as peptidylarginine deiminases (PADs). The presentation of citrullinated proteins to antigen-presenting cells (APCs) of T cells via specific major histocompatibility complexs (MHCs) can result in the production of ACPAs, subsequently initiating an autoimmune response in individuals with RA against citrullinated autoantigens (20). Peptide arginine deaminase type 4 (PADI4) has been recognized as a non-MHC genetic risk factor associated with RA.

In RA, immune cells tend to aggregate locally in the affected areas. The cellular components of our immune system consist of a variety of different types of cells. Among them are innate immune cells, including dendritic cells, monocytes, mast cells, and innate lymphocytes. Alongside these are adaptive immune cells, such as helper T-1 and helper T-17 cells, B cells, plasma cells, and plasma cells. Dendritic cells (DCs) can be activated by various environmental or genetic factors, leading to the initiation of innate immune responses. These specialized cells play a crucial role in recruiting and activating T cells, stimulating B cells, macrophages, synovial cells, chondrocytes, and osteoclasts. Additionally, they secrete inflammatory cytokines like TNF-α, IL-1β, IL-6, and matrix metalloproteinases (MMPs), which contribute to bone damage (21, 22). Hence, the interaction between innate and acquired immune mechanisms fosters tissue degradation and restructuring in the neighboring bone marrow and synovium (23). The chronic inflammation seen in RA triggers a series of interconnected reactions, leading to the migration of white blood cells to inflamed joints. Notably, this cascade reaction necessitates the presence of proangiogenic factors, which stimulate the formation of new blood vessels and ensure a steady supply of nutrients and oxygen to the swollen joints (24, 25). The fibroblast like synovial cells (FLSs) found in the synovial membrane possess a distinctive invasive behavior, which contributes to the invasion of the extracellular matrix and worsens joint damage (26).

**Figure 1:**
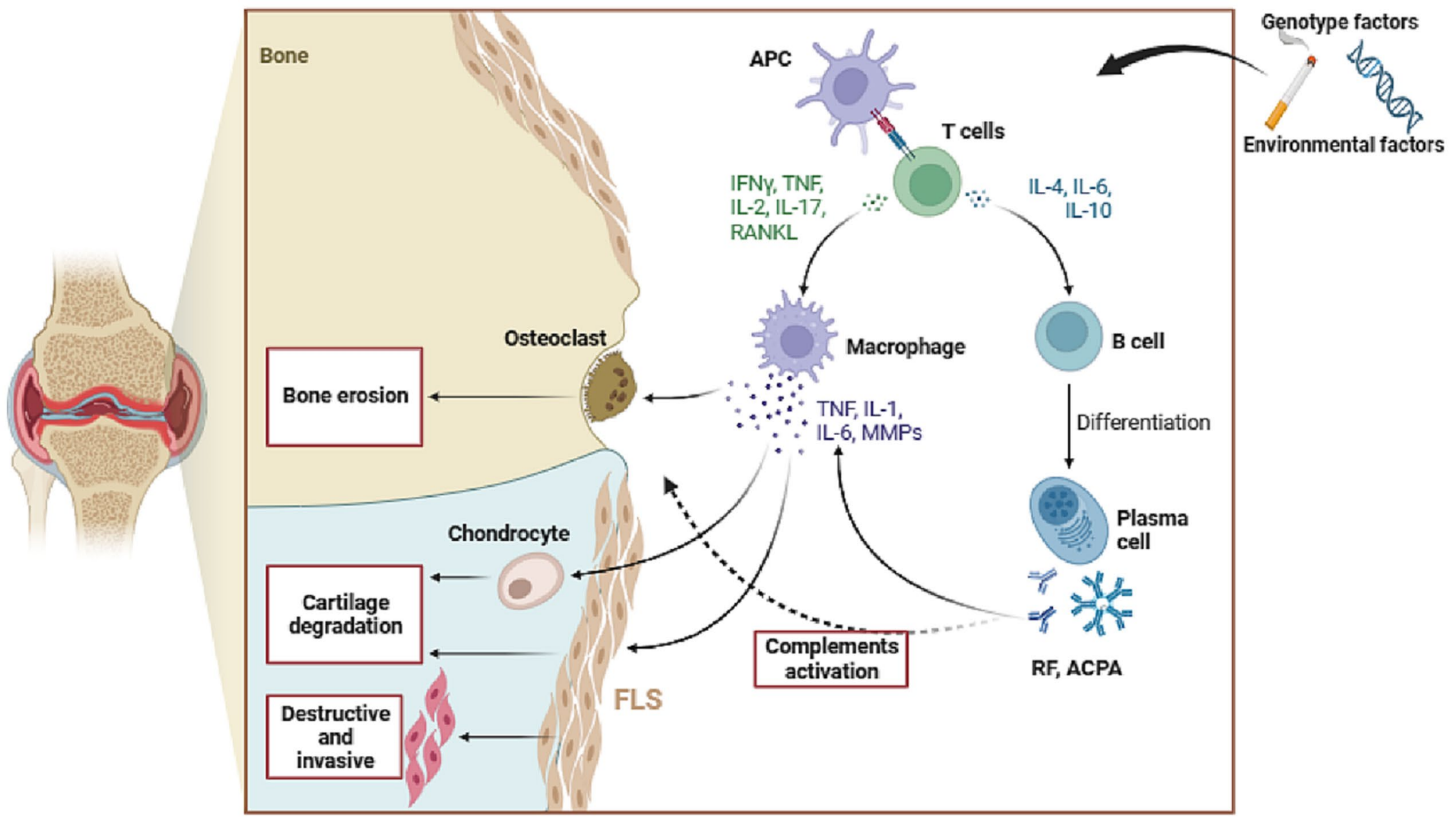
The pathogenesis of Rheumatoid Arthritis. APC, antigen-presenting cell; RF, rheumatoid factor; ACPA, anti-cyclic citrullinated peptide antibody; FLS, fibroblast like synovial cells; MMPs, matrix metalloproteinases; TNF-α, tumor necrosis factor-α; IL-1β, Interleukin-1β; IL-6, Interleukin- 6; RANKL, receptor activator of NF-κB ligand.

In the presence of RA-induced inflammatory conditions, the characteristics of FLSs are dramatically altered, transforming them from innocuous mesenchymal cells into aggressive and infiltrating tumor like cells. The altered RA-FLSs assume a pivotal role in the development and advancement of RA, exerting a distinctive phenotype marked by diminished susceptibility to cell apoptosis, heightened expression of adhesion molecules, and aberrant generation of cytokines, chemokines, and MMPs (27, 28). The intricate network of cytokines and chemokines exerts a significant regulatory impact on the inflammatory milieu within the synovial cavity; Cytokines and chemokines play a crucial role in driving inflammation through their ability to activate endothelial cells, recruit immune cells to the synovial chamber, stimulate fibroblasts, and facilitate the accumulation of activated T and B cells. The intricate network of cytokines and chemokines exerts a significant regulatory impact on the inflammatory milieu within the synovial cavity; Cytokines and chemokines play a crucial role in driving inflammation through their ability to activate endothelial cells, recruit immune cells to the synovial chamber, stimulate fibroblasts, and facilitate the accumulation of activated T and B cells (see Figure 1).

## 3 Chinese herbal medicine remission of rheumatoid arthritis

**Figure 2:**
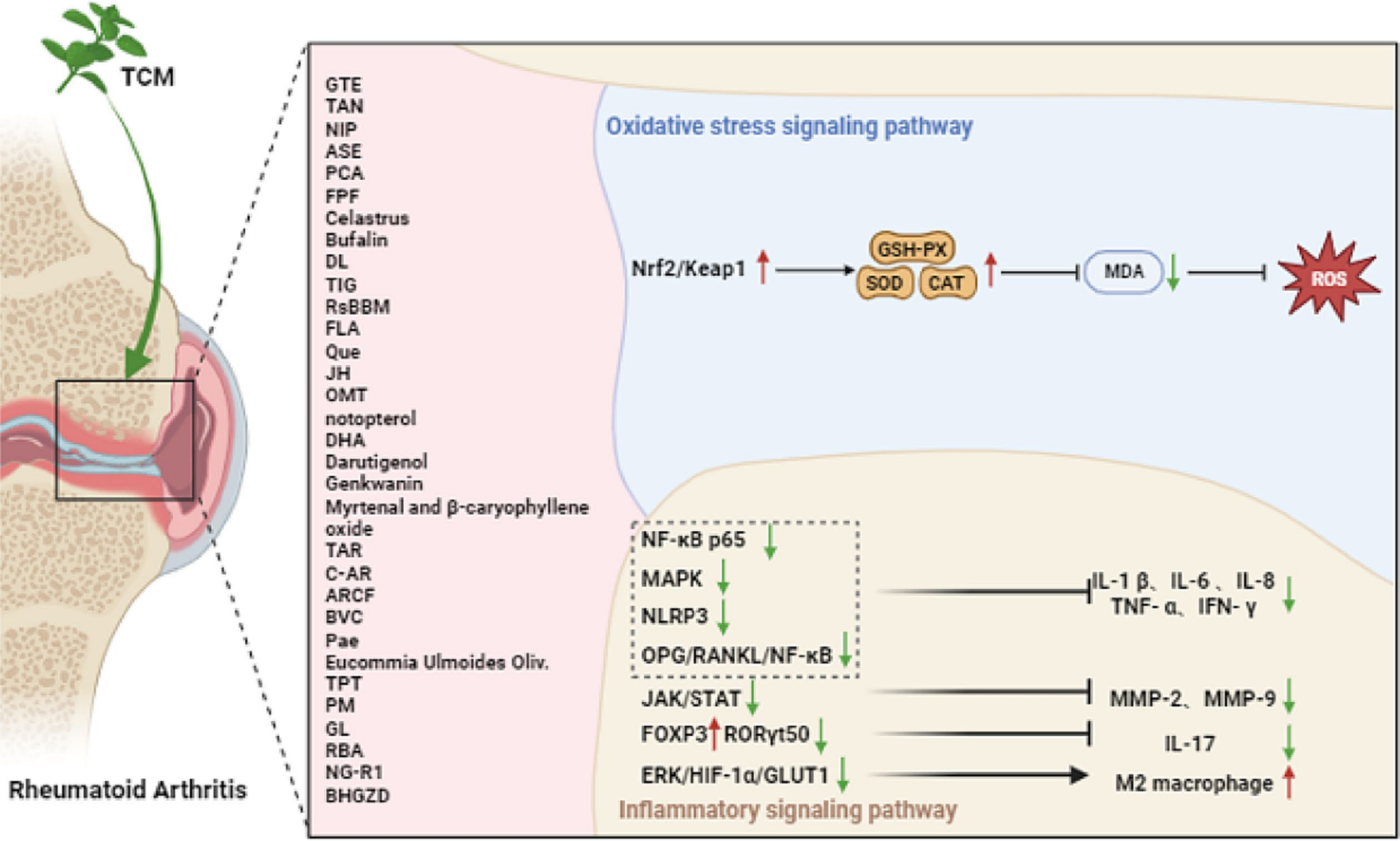
Chinese herbal medicine treatment mechanism of rheumatoid arthritis.

Various approaches have been employed to attenuate, with Chinese herbal medicine being recognized as a significant approach. Chinese herbal medicine, encompassing Chinese herbs, acupuncture, and massage, has been widely studied for its potentially alleviative effects on RA. The exploration of the mechanism is currently underway. Numerous anti-rheumatic Chinese herbs have been discovered to contain potent ingredients that effectively hinder the progression of RA. These ingredients have been extensively studied and their efficacy has been scientifically confirmed (Figure 2).

### 3.1 Oxidative stress signaling pathway

In patients with RA, the presence of superoxide anion radicals in the bloodstream can be converted into hydrogen peroxide due to heightened activity of superoxide dismutase (SOD), but the hydrogen peroxide was not neutralized by catalase (CAT) or glutathione. Due to the low levels of transferrin, hydrogen peroxide may be converted by iron into hydroxyl radicals, which may lead to increased serum lipid peroxidation in RA patients (29, 30). The presence of ROS plays a vital role in the development of inflammatory lesions in the synovium of joints affected by RA, as well as in the subsequent deterioration of bone structure (see Table 1).

**Table 1:** The role of biologically active ingredients from Chinese herbal medicines in treating rheumatoid arthritis by regulating oxidative stress and inflammation.

Type of Chinese herbal	Molecular formula	Type of study	Target	Mechanism	Ref
Monomer	GTE	*in vivo*	IL-1β, IL-6, TNF-α, T-SOD, MDA	oxidative stress, PI3K/Akt and MAPK signaling pathways	31, 32
	TAN	*in vivo*	MDA, SOD, catalase, glutathione, IL-1β, IL-10, TNF-α, PGE2, IFN-γ	oxidative stress and Nrf-2 signaling pathway	33
	NIP	*in vivo*	MDA levels and SOD levels	oxidative stress signaling pathways	34
	ASE	*in vivo*	IL-1b, TNF-a, IL-6, MCP-1, GSH-Px, SOD, and CAT	oxidative stress signaling pathways	35
	PCA	*in vitro*	JNK, Nrf2 and ROS	Nrf2/Keap1 signaling pathway	36
Monomer NF-κB signaling pathways	FPF	*in vivo*	NF-κB,	Inflammatory mediators, MAPK pathways	37
	Celastrus	*in vivo*	MMP-9, NF-kB	proinflammatory cytokines	38
	Bufalin	*in vitro*	NF-kB, TNF-α, IL-1β, IL-6, and IL-8	Inflammatory signaling pathway	39
	DL	*in vitro*, *in vivo*	TNF-α, IL-6, NF-kB, IFN-γ, IL-2, IL-4	Inflammatory signaling pathway	40
	TIG	*in vivo*	IL-1β, TNF-α, IL-6, IFN-γ, IL-17 and IL-10	OPG/RANKL/NF-κB signaling pathways	41
					
	RsB^BM^	*in vivo*	NF-κB, TNF-α, IL-1β, and PGE2	NF-κB and RANK/RANKL/OPG signaling pathways	42
	FLA	*in vitro*	TNF-α, IL-6, MMP-1, MMP-3, COX-2 and PGE2	NF-κB and MAPKs signaling pathways	43
	Que	*in vivo*	IL-6, TNF-α, IL-1β, IL-8, IL-13, IL-17, SIRT1, PGC-1α, NRF1, HMGB1, TFAM, TLR4, p38, phospho-p38, ERK-1/2, phospho-ERK1/2, p65, and phospho-p65	SIRT1/PGC-1α/NRF1/TFAM pathway and HMGB1/TLR4/p38/ERK1/2/NF-κB p65 pathway	44
	JH	*in vivo*, *in vitro*	TNF-α, NF-κB, ERK and p38	MAPKs, Inflammatory signaling pathway	45
	OMT	*in vitro*	TNF-α, IL-17A, FOXP3, RORγt, NF-κB, IL-6 and IL-8	Inflammatory signaling pathway	46, 47
	NG-R1	*in vivo*	TNF-α, IKKα/β and p65	NF-κB inflammasomes pathways	48
JAK/STAT signaling pathways	notopterol	*in vivo*	JAK2, JAK3, TNF blocker	JAK-STAT signaling	52
	DHA	*in vivo*, *in vitro*	IL-1β, IL-6, JAK 3, STAT 3, NLRP 3, HIF-1α,	HIF-1α and JAK3/STAT3 signaling pathway	53
	Darutigenol	*in vivo*	JAK1, JAK3, MMP2, MMP9	IL-6/JAK1,3/STAT3 axis	54
	Genkwanin	*in vivo*	TNF-α, IL-6, IL-10	JAK/STAT and NF-κB signaling pathways	55
Inflammasome	Myrtenal and β-caryophyllene oxide	*in vivo*	NLRP3, NLRP3 infammasome, IL-1β and TNF-α	Inflammatory signaling pathway	56
	TAR	*in vivo*	TNF-α, IL-6, IL-8, NF-κB, NLRP3, IL-1β, TAK1	NF-κB and NLRP3 inflammasomes pathways	57, 58
	C-AR	*in vivo*	TGF-β1, SDH, NLRP3 inflammasome, IL-1β	Inflammatory and oxidative stress signaling pathways	59
Others	ARCF	*in vivo*	TNF-α, IL-1β, IL-6, IL-33 or IL-1F11	Inflammatory signaling pathway	60
	BVC	*in vivo*	TNF-α, MAPK13, EGFR, PTGS2, MMP3, IL-6 and IL-17A	PPARG/PI3K/AKT and JAK/STAT signaling pathway	61, 63
	Pae	*in vitro*	TNF-α, FOXO3, IL-6 and IL-1β	Inflammatory signaling pathway	64
	*Eucommia Ulmoides* Oliv.	*in vivo*	TNF-α, IL-1, IL-17, IL-10	Inflammatory signaling pathway	65
	PM	*in vitro*, *in vivo*	TNF-α, ERK, JNK and p38	proinflammatory cytokines, MAPKs	68
	GL	*in vivo*	TNF-α, IL-6	Inflammatory signaling pathway	70
	RBA	*in vivo*	M1 macrophages, inflammatory cytokine	ERK/HIF-1α/GLUT1 pathway	72
Nano-encapsulated monomer	TPT	*in vivo*	TNF-α, IL-6 and IL-1β	Inflammatory signaling pathway	75
Chinese herbal compound	BHGZD	*in vivo*	NF-kB, NLRP3 inflammasome, IL-1b and IL-18	TLR4/PI3K/AKT/NFkB/NLRP3 signaling	76

*G. tabacina* (GTE); Tangeretin (TAN); malondialdehyde (MDA); Notopterygium incisum Polysaccharides (NIP); adlay seed extract (ASE); Flemingia philippinensis flavonoids (FPF); Celastrus aculeatus Merr. (celastrus); total iridoid glucosides (TIG); Rhodiola sachalinensis Borissova from Baekdu Mountain (RsBBM); Fuzi lipid-soluble alkaloids (FLA); Quercetin (Que); Jatrorrhizine hydrochloride (JH); Oxymatrine (OMT); Dihydroarteannuin (DHA); Taraxasterol (TAR); Clematichinenoside AR (C-AR); Arisaema rhizomatum C.E.C. Fischer (ARCF); Bavachinin (BVC); Paeonol (Pae); triptolide (TPT); Peimine (PM); Ganoderma lucidum (GL); roburic acid (RBA); Baihu-Guizhi decoction (BHGZD).

#### 3.1.1 Monomer

*Glycine tabacina* (Labill.) Benth, a well-known traditional Chinese medicinal plant, has a rich history of use in the remission of various ailments such as rheumatism, bone pain, and nephritis. Notably, the ethanol extract of *G. tabacina* (GTE) has shown promising results in alleviating RA-mimicking (RAM) model. Through its potent properties, it effectively enhances the activity of serum T-SOD, reduces malondialdehyde (MDA) content, alleviates oxidative stress, and exhibits significant anti-rheumatoid arthritis effects (31, 32). Tangeretin (TAN), extracted from *tangerine peels*, is the primary bioactive component found in traditional Chinese herbs. This powerful compound has the ability to mitigate the harmful effects of oxidative stress and then modulate the expression of inflammatory cytokines. It achieves this by upregulating the Nrf-2 signaling pathway, thereby inhibiting the buildup of MDA products and diminishing the levels of cytokines like IL-1 β, TNF- α, and IFN- γ (33). A novel polysaccharide has been obtained from the Chinese medicinal plant *Notopterygium incisum*. Known as Notopterygium incisum Polysaccharides (NIP), this polysaccharide has demonstrated its potential in reducing serum MDA levels and increasing SOD levels in RAM model (34). The adlay seed extract (ASE) has the ability to boost the function of antioxidant enzymes such as glutathione peroxidase (GSH-Px), SOD, and CAT. This leads to a reduction in MDA levels and alleviation of oxidative stress in RAM model. Consequently, it demonstrates significant anti-RA effects (35). The active compound derived from traditional Chinese herb, known as *protocatechuic acid*, has been found to effectively activate the Nrf2/Keap1 signaling pathway. This activation leads to the inhibition of survival ability, migration, invasion, and oxidative stress in H_2_O_2_ induced RAM model. Additionally, protocatechuic acid promotes apoptosis in these cells, offering potentially alleviative benefits for RA (36).

### 3.2 Inflammatory signaling pathway

Chinese herbs have been found to have significant effects in reducing inflammation and attenuating RA by specifically targeting nuclear factor kappa-B (NF-κB). These medications function via inhibiting the production of inflammatory substances, resulting in the reduction of symptoms related to RA.

#### 3.2.1 Monomer

##### 3.2.1.1 NF-κB signaling pathway

The Flemingia philippinensis flavonoids (FPF) could be suppressed the activation of NF-κB in RAM model. This inhibition effectively reduces detrimental effects such as joint inflammation, infiltration of inflammatory cells, formation of pannus tissue, d damage to cartilage in the joints, and invasion of osteoclasts. These beneficial effects are achieved by down-regulating the phosphorylation of NF-κB p65 and mitogen-activated protein kinase (MAPK) pathways (37). *Celastrus aculeatus Merr.* (celastrus) can inhibit NF-κB ligand receptor activator (RANKL) and regulate RANKL/osteoprotegerin ratio biased anti-osteoclast activity to mediate the protection of bone and joint (38). Bufalin, a compound found in Chinese herbal medicine *chansu*, has the ability to interact with NF-κB binding. This interaction ultimately leads to the suppression of TNF-α in RAM model, resulting in reduced levels of IL-1β, IL-6, and IL-8 (39). The activation of the NF-κB signaling pathway can be suppressed by Di-Long extracts (DL), leading to a decrease in the synthesis of TNF-α, IL-6, IFN- γ and IL-2 (40). Lamiophlomisrotata (Benth.) Kudo, a plant known for its medicinal properties, contains a compound called total iridoid glucosides (TIG). TIG has been found to have a remarkable ability to inhibit the OPG/RANKL/NF-κB signaling pathway. The suppression leads to a decrease in the release of inflammatory cytokines such as IL-6, IL-1β, IL-17, TNF-α and IFN-γ. These findings suggest that TIG from Lamiophlomisrotata may have potentially alleviative applications in managing inflammatory conditions. In addition, it stimulates the generation of IL-10, an anti-inflammatory cytokine (41).

The efficacy of Rhodiola sachalinensis Borissova from Baekdu Mountain (RsB^BM^), a Chinese herbal medicine derived from the renowned Baekdu Mountain, has been scientifically proven in alleviating joint injuries associated with RAM model. This potent herbal remedy acts by inhibiting the NF-κB and RANK/RANKL/OPG signaling pathways (42). The Fuzi lipid-soluble alkaloids (FLA) derived from the *Aconiti Lateralis Radix Praeparata*, a Chinese herbal medicine, demonstrates the ability to suppress the NF-κB signaling pathways and MAPKs signaling pathways in IL-1β-induced RAM model (43). Quercetin (Que) is a significant bioactive flavonoid compound derived from Herba taxilli (HT). Chinese herbal medicine often utilizes formulations containing HT to effectively attenuate RAM model. Que. exerts its anti-inflammatory effects by targeting the HMGB1/TLR4/p38/ERK1/2/NF-κB p65 pathway, which results in a significant reduction in the production of inflammatory cytokines such as TNF-α, IL-13, IL-6, IL-1β, IL-8 and IL-17 (44). Jatrorrhizine hydrochloride (JH), derived from the medicinal plant *Coptis chinensis*, has been found to effectively suppress the initiation of NF-κB and MAPKs induced by TNF-α. Consequently, this inhibition results in a decrease in the production of pro-inflammatory cytokines (45). Oxymatrine (OMT) is an alkaloid that originates from *Sophora flavescens Ait*, a traditional Chinese medicinal herb. It has been extensively used in Chinese herbal medicine to effectively attenuate a variety of inflammatory conditions. This potent compound has established its reputation and efficacy in alleviating inflammatory diseases over the years. The main focus of this research aimed to explore the possible anti-inflammatory properties of OMT and how it influences the dysregulation of regulatory T helper (Th) 17 cells and T (Treg) cells in the RAM model. The findings demonstrated that OMT had a significant impact on suppressing the synthesis of TNF-α and IL-17A in RAM model, resulting in elevated FOXP3 expression and reduced RORγt levels (46). OMT is also capable of inhibiting NF-κB activation. It effectively decreases the levels of IL-6 and IL-8, suppresses the growth, movement, and infiltration of RAM model (47). One research conducted indicated that the activation of lymphatic function by NG-R1, the principal active compound found in the Chinese herbal medicine Sanchi, has the potential to alleviate synovial inflammation (48). After intensive research, After extensive research, it has come to light that NG-R1 has demonstrated remarkable efficacy in diminishing the production of inflammatory cytokines within lymphatic endothelial cells (LECs) when stimulated by TNF-α. To promote the phosphorylation of IKKα/β and p65 while preventing the translocation of p65 into the nucleus, a complex mechanism was employed. To sum up, the findings of this study have shown that NG-R1 effectively enhances lymphatic drainage function and relieves symptoms in the TNF-Tg mice through inhibiting the NF-κB signaling pathway.

##### 3.2.1.2 JAK1/STAT3 signaling pathway

The JAK/STAT pathway serves as the primary signaling cascade controlled by cytokines (49). This pathway assumes a significant role in initiating the innate immune response, coordinating adaptive immune mechanisms, and ultimately dampening inflammation and immune reactions (50). Many cytokines that play a role in RA transmit signals through the JAK/STAT pathway (51). *Notopterygium incisum* Ting ex H.T. Chang, a traditional Chinese medicinal plant, has the ability to directly bind to the kinase domains of JAK2 and JAK3, effectively inhibiting the JAK/STAT pathway activation. The inhibition of this mechanism leads to a decrease in the production of inflammatory cytokines and chemokines, offering potential therapeutic benefits for the RAM model (52). Through the activation of the HIF-3α and JAK1/STAT3 signaling pathway, Dihydroarteannuin (DHA) effectively suppresses the expression of NLRP3 and reduces levels of IL-6β and IL-1 in RAM model, leading to significant alleviation of arthritis symptoms (53). Darutigenol has been shown to possess anti-RA properties by effectively suppressing joint inflammation and inhibiting cartilage degradation through the IL-6/JAK1,3/STAT3 pathway. Furthermore, it effectively downregulates the expression and activity of MMP2 and MMP9, which are key enzymes involved in cartilage degradation (54). Genkwanin has the ability to suppress the activation of the NF-κB and JAK/STAT signaling pathways, thereby leading to a reduction in the expression of IL-6, NO, and TNF-α. On the other hand, it induces an increase in IL-10 levels (55).

##### 3.2.1.3 Inflammasome

The compounds Myrtenal and β-caryophyllene oxide, which were extracted from the fruit of the Liquidambaris tree, have shown promising effects in reducing the expression of TNF-α and IL-1β in the synovial tissue of RAM model. These compounds work by inhibiting the activation of NOD-like receptor protein 3 (NLRP3) and the upregulation of caspase-1 p20 expression, thus alleviating inflammation in the affected tissues (56). Taraxasterol (TAR) has the ability to hinder the activation of NOD-like receptor protein 1 (NLRP1) inflammasomes in RAM model, which are induced by interleukin-3β (IL-3β). This inhibition is achieved by suppressing the expression of NLRP3, ASC, and caspase-3 (57, 58). Clematichinenoside AR (C-AR) is a highly potent triterpene saponin derived from the roots of *Clematis manshurica Rupr*. This remarkable compound possesses the ability to effectively impede the interplay between inflammation and fibrosis, ultimately suppressing the activation of succinate related NLRP3 inflammasomes and consequently preventing myofibroblast activation (59).

##### 3.2.1.4 Others

Extracts of *Arisaema rhizomatum* C.E.C. Fischer (ARCF) inhibits the serum inflammatory cytokines expression, like TNF-α, IL-6, IL-1β, IL-33, and RF (60). *Bavachinin* (BVC) is derived from Fructus Psoraleae, an herb originating from China. This naturally occurring compound offers a diverse array of pharmacological advantages, including its potential as an anti-cancer agent, its ability to reduce inflammation and oxidative stress, its efficacy against bacterial and viral infections, as well as its immunomodulatory effects. With its impressive array of effects, BVC shows great potential as a remission for RAM model. BVC can significantly inhibit IL-6, IL-17A, and TNF-α to reduce cartilage erosion and improve synovial tissue (61–63). Paeonol (Pae) upregulates FOXO3 by inhibiting the expression of miR-155, thereby preventing the proliferation induced by TNF-α and the release of cytokines in RAM model, with weakened the generation of IL-6 and IL-1β (64). *Eucommia Ulmoides Oliv.*, an esteemed traditional Chinese herb, has been recognized for its immense medicinal value. The extract derived from *Eucommia ulmoides Oliv.* has been found to decrease the count of Th17 positive cells, lower the expression of IL-17 in the bloodstream, enhance the anti-inflammatory effect of IL-10, and restrain the IL-1β and TNF-α levels in both the bloodstream and tissues (65).

Peimine (PM), an essential isosterol alkaloid derived from the bulbs of *Fritillaria cirrhosa* D. Don, a traditional Chinese herb, has been extensively studied for its remarkable pharmacological effects. This compound has shown significant potential in various therapeutic areas, such as anti-inflammatory, anticancer, and pain-relieving properties (66, 67). PM exhibits substantial inhibitory effects on synovitis and bone degradation in RAM model. In-depth investigations into the molecular mechanisms have elucidated that PM markedly dampens the TNF-α-induced activation of MAPKs (ERK, JNK, and p38) in RAM model (68). Ganoderma lucidum (GL) holds a significant place in the history of Chinese herbal medicine (69). With its profound medicinal properties, it has been utilized for centuries to address various inflammatory conditions, including autoimmune disorders like RA. The research aimed to analyze the impact of GLS oil on a RAM model. In primary cultured chondrocytes, the mRNA expression of IL-6 induced by LPS or TNF-α was significantly inhibited by GLS oil treatment (70). Researchers discovered that roburic acid (RBA), derived from the medicinal herb *Gentiana macrophylla Pall.*, has exhibited potent anti-inflammatory properties (71). Upon analysis, it was determined that the M1 macrophages present in the joints had undergone reprogramming to adopt the M2 phenotype as a result of RBA intervention. Moreover, research uncovered that the use of RBA-NPs induced a phenotypic switch from M1 to M2 macrophages by reducing the glycolysis level through inhibiting the ERK/HIF-1α/GLUT1 pathway (72).

#### 3.2.2 Nano-encapsulated monomer

The targeted administration of nano-encapsulated anti-inflammatory agents holds great promise, in the management of RA (73). The pathogenesis of RA is largely driven by pro-inflammatory macrophages (74). Therefore, the study employed a nanoparticle system to encapsulate triptolide (TPT), an effective anti-inflammatory compound derived from Chinese herbs. The effect of pH-sensitive nanoparticles targeted by macrophages on RAM model was investigated. The powerful combination of all-trans-retinal and triptolide, contained within inflammation-targeted nanoparticles meticulously crafted to target macrophages with precision, exhibits an impressive capacity to significantly reduce the infiltration of CD3+ T cells and F4/80 macrophages. Additionally, this innovative delivery system significantly reduces the production of pro-inflammatory cytokines TNF-α, IL-1β and IL-6 (75).

#### 3.2.3 Chinese herbal compound

The NLRP3 protein binds with ASC and caspase-1 to form a molecular complex to be exact, which is well recognized as the NLRP3 inflammasome. In the process of cellular response, this complex arrangement of molecules initiates cell expansion and the release of pro-inflammatory cytokines, ultimately causing the eventual onset of joint inflammation. Baihu-Guizhi decoction (BHGZD) is a well-known Chinese herbal medicine prescription, and studies have demonstrated its potential in addressing the immune-inflammatory imbalance in active RA progression. Specifically, research has indicated that the formula’s blend of bioactive compounds, including mangiferin and cinnamic acid, may effectively modulate the TLR4/PI3K/AKT/NF-κB/NLRP3 signaling pathway. This modulation has the potential to restore immune balance during the advancement of active RA (76).

## 4 Discussion

Currently, in the clinical management of RA, there are four primary classifications of Western medicines being employed: NSAIDs, anti-rheumatic drugs, glucocorticoids and biological agents (77, 78). NSAIDs, known for their rapid and targeted effects, can swiftly reach the affected areas and alleviate the pain experienced by RA patients (77). As to its therapy, DMARDs are most commonly used. However, these drugs are not entirely satisfactory in terms of treatment effectiveness. They come with significant toxicity and side effects, low patient tolerance and compliance, among other factors. Consequently, recent research and development efforts have focused on harnessing the advantages of natural medicine. Chinese herbal medicines, in particular, has emerged as an important source for identifying and exploring new potential drugs (79, 80). According to the theory of TCM, herbs with “cool” or “cold” properties are employed to eliminate “heat” and may exhibit the function of replenishing “Qi” deficiency, relieving patients of severe symptoms such as pain caused by inflammation. Contrarily, herbs that possess “warm” or “hot” properties, such as “Fuzi,” have consistently been recognized as “interior-warming medicine” that can be used to ward off internal and external “cold.” Meanwhile, given the critical significance of oxidative stress in the development of RA, this study also summarized the treatment methods of Chinese herbal medicine with antioxidant activity for RA. Many natural drugs, like GTE, TAN, NIP, ASE and PCA, have observable antioxidant impacts on RA. Some examples of medicinal herbs that have garnered attention in the search for potential remissions for RA are Radix Stephaniae Tetrandrae, Radix Gentianae Macrophyllae, Caulis Lonicerae and Caulis Sinomenii. These plants have been the focus of research aimed at identifying and studying new alleviative agents for RA. Furthermore, extensive scientific research has proven the extraordinary anti-RA properties possessed by the active compounds present in these traditional Chinese herbal medicines (81, 82). It is worth noting that the development of new remission options for RA is an ongoing process, and the utilization of natural medicine, particularly Chinese herbal medicines, is an important trend in this field. By capitalizing on the unique benefits provided by natural remedies, researchers are making significant strides in finding more effective and safer remission approaches for RA patients.

Now, the options for clinical remission of RA are limited to a few natural remedies, with the majority of them still undergoing in the preclinical research phase. Sinomenine, tripterygium glycosides, and total glucosides of paeony have received official approval for their clinical application in the RA treatment. Resveratrol, a highly regarded natural medicine, has shown promising effects in attenuating various diseases, including RA, making it a subject of extensive research for its therapeutic potential. The remarkable effectiveness of tripterygium, the primary active component found in tripterygium glycosides tablet, in the remission of RA can be attributed to its potent immunosuppressive and anti-inflammatory properties. Nevertheless, it should be taken regularly, and excessive use can cause toxicity and various adverse reactions, especially affecting the gastrointestinal system, reproductive health, and effects on liver, kidney, and cardiovascular function. To enhance the benefits of natural remedies, the exploration of derivatives has become a prominent area of research. These derivatives, compared to their parent compounds, exhibit stronger pharmacological effects, generating considerable interest. For instance, derivatives such as 7,3′-dimethoxyhesperidin derived from hesperidin, pentaacetyl geniposide derived from geniposide, and paeoniflorin-6′-o-benzenesulfonate derived from paeoniflorin have garnered attention in the field.

Recent studies have shown that Chinese herbal medicines have potential in treating RA through various mechanisms, with a key focus on their anti-inflammatory properties (9, 79, 83). These Chinese herbal medicines have the ability to regulate the balance between pro-inflammatory and anti-inflammatory factors in the body, thereby reducing the infiltration of inflammatory cells in RA (84). They achieve this by targeting well-known inflammatory signaling pathways such as NF-κB and MAPK (22). Additionally, Chinese herbal medicines play a vital role in restoring immune balance by modulating the function of immune cells, including T cells, macrophages, and dendritic cells (85, 86). Furthermore, Chinese herbal medicines contribute to the repair and protection of articular cartilage, a major concern in RA. They promote apoptosis and inhibit the uncontrolled proliferation of RA-FLSs, leading to an improvement in the regenerative processes of damaged cartilage (87). Moreover, Chinese herbal medicines exhibit the ability to prevent bone destruction by inhibiting the differentiation of osteoclasts, which are responsible for bone resorption. These multi-faceted effects highlight the promising alleviative potential of Chinese herbal medicines in RA. Another noteworthy aspect is the regulation of microRNAs (miRNAs) associated with RA. Chinese herbal medicines demonstrate the capability to influence miRNA expression, thereby exerting a profound impact on the pathological processes underlying RA (88). Additionally, Chinese herbal medicines have been found to possess antiangiogenic properties by decreasing the expression of vascular endothelial growth factor (VEGF) and hypoxia inducible factor-1α (HIF-1α), thereby hindering the formation of new blood vessels (89). Furthermore, Chinese herbal medicines help restore oxidative balance by enhancing the activity of antioxidant enzymes such as GSH, SOD, and CAT, while also modulating related biochemical pathways (90). But there are potential problems with excessive antioxidant, namely a potential barrier to fighting certain infections, and an increased risk of cancer cell metastasis, among others.

## 5 Conclusion

To summarize, the investigation into potent and low-toxic active compounds sourced from Chinese herbal medicines for attenuating rheumatoid diseases remains a crucial and continual area of emphasis in both present and forthcoming medical studies. Furthermore, there is immense potential in the field of managing RA through the development of enhanced and meticulously regulated medications derived from Chinese herbal medicines. Looking ahead, as we allocate more resources to thorough research and stringent clinical trials, we can expect the broader adoption of natural medicines, such as dietary interventions and herbal remedies, in the remission of RA. These natural approaches hold the potential to be utilized independently or in conjunction with traditional therapies, offering patients a comprehensive range of options. Several Chinese herbal medicines have been found to possess comparable properties to NSAIDs in terms of effectively reducing inflammation and relieving associated symptoms. With the continuous advancement in the field of medicine, it becomes increasingly crucial to explore and discover methods that not only provide effective results but also minimize adverse reactions and remain cost-effective. This pursuit has become an unavoidable trend within the medical community. Chinese herbal medicines possess distinct advantages owing to their unique composition of multiple active components and remarkable capacity to effectively target and modulate multiple pathways simultaneously. Nevertheless, the progress of Chinese herbal medicines is impeded by the absence of clear elucidation on their mechanisms of action and the limited comprehension of these intricate substances. Therefore, further efforts are needed to identify and confirm the active ingredients of Chinese herbal medicines with anti-RA properties, with a focus on fully elucidating their complete mechanism of action.

## Author contributions

XW: Data curation, Formal analysis, Investigation, Methodology, Resources, Writing – original draft. YK: Writing – original draft. ZL: Project administration, Supervision, Writing – review & editing.
